# Reductive Al−B σ‐Bond Formation in Alumaboranes: Facile Scission of Polar Multiple Bonds

**DOI:** 10.1002/anie.202209502

**Published:** 2022-09-02

**Authors:** Zeynep Güven, Lars Denker, Daniela Wullschläger, Juan Pablo Martínez, Bartosz Trzaskowski, René Frank

**Affiliations:** ^1^ Department of Inorganic and Analytical Chemistry Technische Universität Braunschweig Hagenring 30 38106 Braunschweig Germany; ^2^ Centre of New Technologies University of Warsaw Banacha 2 C 02-097 Warsaw Poland

**Keywords:** Alumaborane, Bond Scission, Migration Reactions, Polarized Bonds, σ-Bonds

## Abstract

We present facile access to an alumaborane species with electron precise Al−B σ‐bond. The reductive rearrangement of 1‐(AlI_2_), 8‐(BMes_2_) naphthalene (Mes=2,4,6‐Me_3_C_6_H_2_) affords the alumaborane species *cyclo*‐(1,8‐C_10_H_6_)‐[1‐Al(Mes)(OEt_2_)‐8‐B(Mes)] with a covalent Al−B σ‐bond. The Al−B σ‐bond performs the reductive scission of multiple bonds: S=C(N*i*PrCMe)_2_ affords the naphthalene bridged motif B−S−Al(NHC), NHC=*N*‐heterocyclic carbene, while O=CPh_2_ is deoxygenated to afford an B−O−Al bridged species with incorporation of the remaining ≡CPh_2_ fragment into the naphthalene scaffold. The reaction with isonitrile Xyl‐N≡C (Xyl=2,6‐Me_2_C_6_H_4_) proceeds via a proposed (amino boryl) carbene species; which adds a second equivalent of isonitrile to ultimately form the Al−N−B bridged species *cyclo*‐(1,8‐C_10_H_6_)‐[1‐Al(Mes)‐N(Xyl)‐8‐B{C(Mes)=C−N−Xyl}] with complete scission of the C≡N triple bond. The latter reaction is supported with isolated intermediates and by DFT calculations.

## Graphical abstract

The formation of rare Al−B σ‐bonds is facilitated employing a *peri*‐AlI_2_/BMes_2_ naphthalene scaffold, which upon reduction forms an alumaborane diethyl ether adduct. Reagents with polar multiple bonds (C=O, C≡N, C=S) undergo complete scission with insertion of the heteroatom into Al−B bond. The findings help to understand the differences between Al−C bonds widely employed in aluminum organyls and scarcely studied alumaboranes with Al−B bonds.

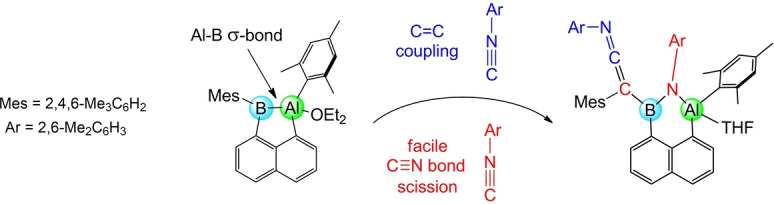

## Introduction

**Scheme 1:**
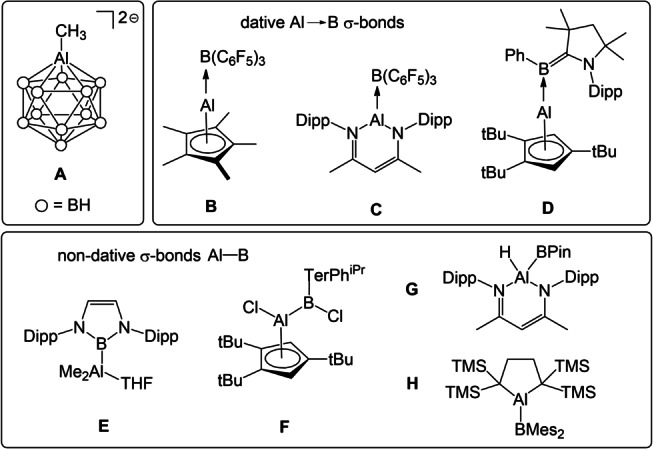
Summary of reported species with covalent Al−B bonds. Cations omitted for clarity. Dipp=2,6‐iPr_2_C_6_H_3_. Mes=2,4,6‐Me_3_C_6_H_2_.

Aluminum is the third most abundant element in Earth's crust and occurs predominantly in the form of oxide and silicate minerals. The availability, low cost and nontoxicity of this element have widely stimulated early applications of its compounds, which especially holds for aluminum organometallic reagents on laboratory and industrial scale.^[1]^ Since the early discovery of the first organoaluminum compound Et_3_Al_2_I_3_, produced from elemental aluminum and ethyl iodide in 1859,^[2]^ continuous development in this realm has been achieved, which is well documented by the growing number of papers, patents, and books.^[3]^ The formation of the inherent Al−C bond in organoaluminum compounds is commonly facile, and synthetic approaches include i) oxidative addition of organic halides to elemental aluminum, ii) salt metathesis of lithium or magnesium organyls with aluminum halides, and iii) reactions of mercury organyls with elemental aluminum in transmetallation reactions. In contrast, for boron ‐ carbon′s neighbor in the periodic table ‐ such a general methodology is absent, which accounts for the currently limited number of representative compounds with Al−B bonds and the lack of understanding of their chemical reactivity. In the past, borane or carborane clusters were exploited to establish the first examples of Al−B bonds with (at least partly) covalent character as found in anion **A** (Scheme 1). The bonding is governed by Wade's rules leading to electron non‐precise Al−B bonds.^[4]^ In contrast, compounds that display electron precise covalent Al−B bonds are extremely rare, and only a handful of examples such as **B−H** have been reported. Thus, compounds **B−D** were formed in reactions of Al^I^‐nucleophiles with Lewis acidic boranes,^[5]^ and the interaction between the two elements can be well described in view of a σ‐dative Al→B bond character. Likewise, the number of compounds with non‐dative Al−B σ‐bond such as **E−H** is very limited. The access to alumaboranes of type **E** was facilitated by reactions of nucleophilic, anionic boron reagents^[6]^ with aluminum electrophiles as demonstrated by Anwander^[7]^ and Kinjo.^[8]^ With the inverted reagent functionality, neutral Al^I^ nucleophiles reacted with boron electrophiles with formation of the Al−B bond as demonstrated by Braunschweig (**F**)^[9]^ and Nikonov (**G**).^[10]^

A very recent example reported by Yamashita employed an anionic aluminum nucleophile with a boron electrophile to produce alumaborane **H**.^[11]^ Despite the importance of the reported compounds **B−H** in the chemistry of boron, the reactivity of electron precise Al−B‐bonds has only marginally been studied. Only for the recent alumaborane **H**, reactions with dimethyl sulfoxide and carbon monoxide gave deoxygenation of these two substrates with concomitant formation of the Al−O−B structural motif.

## Results and Discussion

**Scheme 2:**
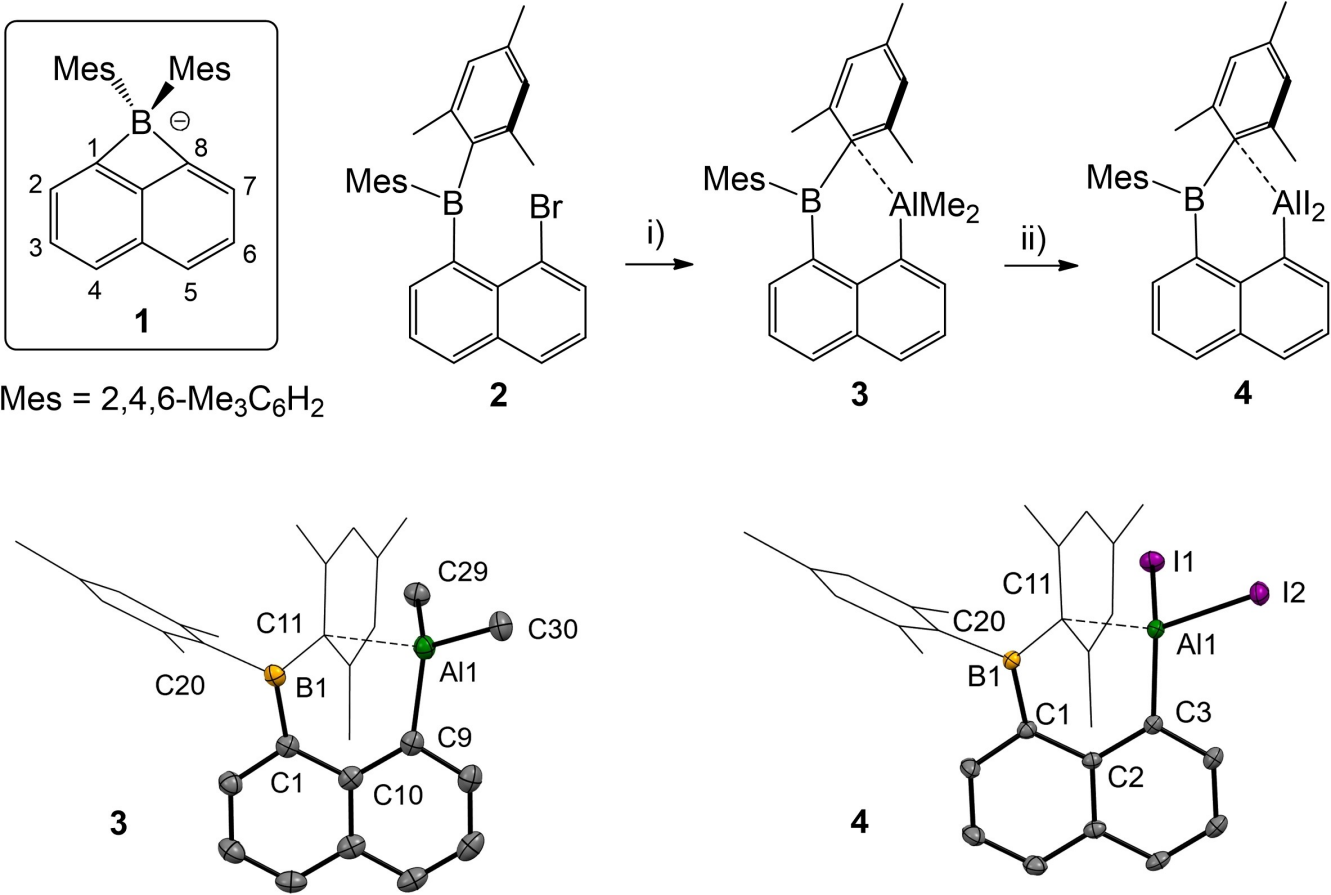
Synthesis of precursor **4**. Cations for **1** omitted. Reagents and conditions. i) 2.0 eq. tBuLi, Et_2_O, −78 °C, 30 min, then 1.0 eq. AlMe_2_Cl, rt, overnight, 60 %. ii) 1.2 eq. AlI_3_, PhMe, rt, 3 h, 90 %. Molecular structures of compounds **3** and **4** with omitted hydrogen atoms and thermal ellipsoids presented at 50 % probability. Selected bond lengths (−) and contacts (⋅⋅⋅) in Å and bond angles in deg. For **3**: Al1−C9 1.9755(16), Al1⋅⋅⋅C11 2.4092(14), B1−C1 1.560(2), B1−C11 1.603(2), C30−Al1−C9 114.39(7), C9−Al1⋅⋅⋅C11 94.84(5), C1−B1−C11 126.00(12). For **4**: I1−Al1 2.5311(11), I2−Al1 2.5172(11), Al1−C3 1.951(3), Al1⋅⋅⋅C11 2.232(3), C1−B1 1.565(4), C1−B1−C11 125.6(3), C3−Al1⋅⋅⋅C11 100.55(13), I2−Al1−I1 107.10(4). Mes=2,4,6‐Me_3_C_6_H_2_.

Our approach to systematic Al−B bond formation was inspired by previous work by Gabbaï, who developed convenient access to aryl borate anion **1** with a BMes_2_ moiety in a bridging position of the *peri*‐disubstituted naphthalene scaffold (Scheme 2).^[12]^ The inherent strain in the cyclic four‐membered borate anion **1** facilitated ring opening reactions with electrophiles and allowed for the synthesis of *peri*‐disubstituted heteronuclear B/Ga and B/In naphthalene derivatives.^[12b]^ Despite the well‐studied chemistry of anion **1**, disubstituted *peri*‐Al/B naphthalene derivates have never been reported. We considered the *peri*‐disubstituted naphthalene scaffold as a suitable template to preorganize the elements aluminum and boron in a spatial vicinity for the enforcement of covalent Al−B bonds. Thus, we commenced our study with the literature known starting material **2** ^[13]^ and introduced the aluminyl group AlMe_2_ via the lithiation ‐ salt elimination procedure. The resulting heteronuclear Al/B naphthalene **3** was reacted subsequently with aluminum‐(III) iodide, which afforded derivative **4** with the diiodoaluminyl moiety AlI_2_. While the ^1^H NMR spectra of **2** at ambient temperature showed broad signals due to inherent dynamic behavior, the spectra of **3** and **4** display well resolved narrow signals.

**Scheme 3:**
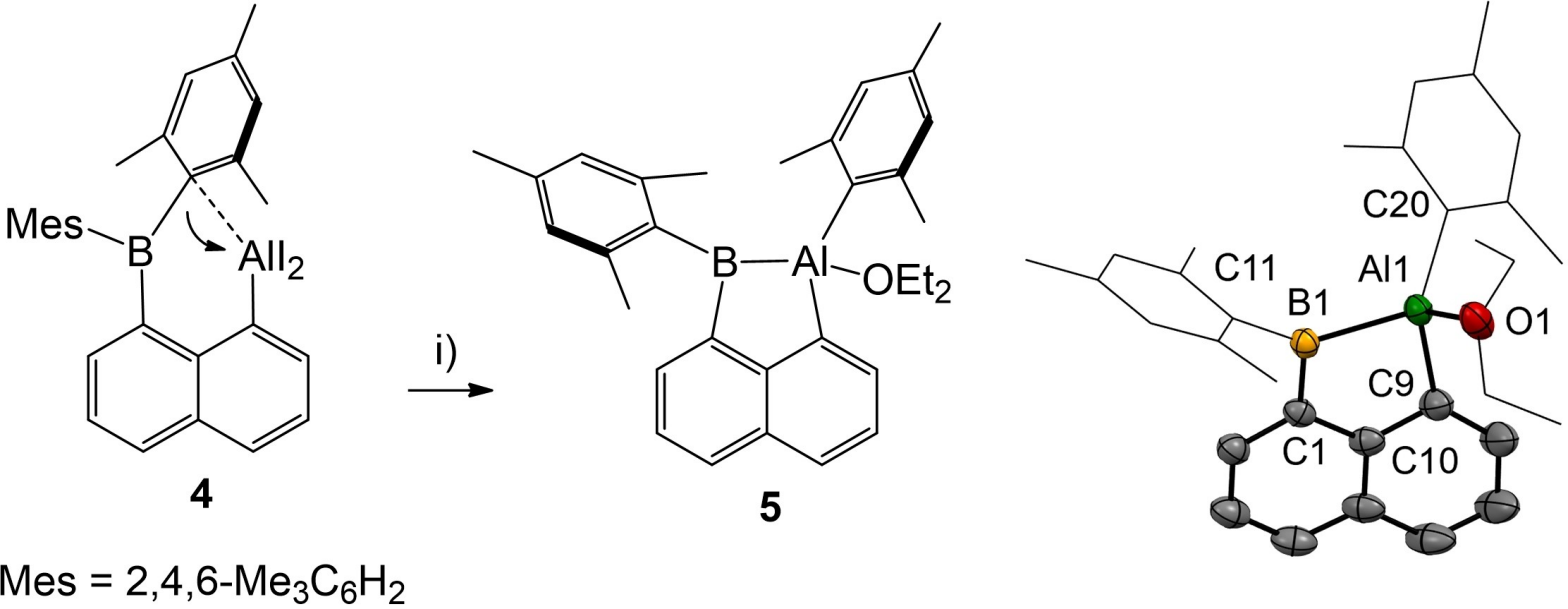
Synthesis of compound **5**. Reagents and conditions. i) 2.1 eq. KC_8_, Et_2_O, rt, 60 min, 65 %. Molecular structure of compound **5** with omitted hydrogen atoms and thermal ellipsoids presented at 50 % probability. Selected bond lengths in Å and bond angles in deg. Al1−O1 1.9183(15), Al1−C9 2.016(2), Al1−C20 2.0045(18), Al1−B1 2.1481(19), C1−B1 1.564(2), C11−B1 1.569(2), C9−Al1−B1 88.72(8), C10−C9−Al1 109.44(13), C10−C1−B1 118.34(15), C1−B1−Al1 102.23(11).

Furthermore, the ^1^H NMR spectra strongly suggest C_1_ symmetry for both **3** and **4** in solution. In particular, the mesityl groups indicate chemical inequivalence and give rise to six well‐separated methyl singlets between δ=1.00–2.50 ppm. The ^11^B{^1^H} NMR spectra of compounds **2**–**4** (δ=70.0–75.0 ppm) are less diagnostic, and the ^11^B nucleus does not respond significantly upon the introduction of the aluminyl entities. The origin of the observed C_1_ symmetry for **3** and **4** is rationalized from X‐ray crystallographic analyses (Scheme 2). In both compounds **3** and **4** close contact interactions Al1⋅⋅⋅C11 of the Lewis acidic aluminum center Al1 with the *ipso*‐carbon atom C11 of one adjacent mesityl group are unambiguously found. In particular, in compound **4** the contact Al1⋅⋅⋅C11 [2.232(3) Å] is only ca. 0.3 Å longer than the obviously covalent bond in Al1−C3 [1.951(3)] found in the same molecule. Similar contact interactions have already been reported by Gabbaï for the heavier group 13 elements gallium and indium albeit to a lower extent, which can be rationalized based on the stronger Lewis acidic behavior of aluminum.^12b^ In accordance with the intramolecular contacts, the Al1 centers in both **3** and **4** deviate from the trigonal planarity and adopt a well‐shaped tetrahedral geometry. In view of the short Al1⋅⋅⋅C11 contact and the excellent fugacity of iodido ligands, compound **4** was considered a promising precursor for the covalent Al−B bond formation by reductive routes (Scheme 3). Thus, the reaction of **4** with potassium graphite (2.1 eq.) in diethyl ether afforded compound **5** in 65 % yield as a magenta‐colored powder. The formation of the Al−B σ‐bond in **5** is accompanied by (intramolecular) migration of a mesityl entity from boron to aluminum, which can be rationalized based on the preorientation and the contact interaction between the aluminum center and the *ipso*‐carbon atom of a mesityl group as observed in the starting material **4**.

**Scheme 4:**
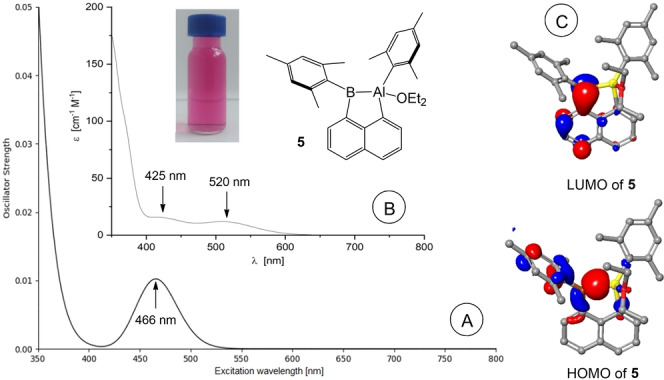
A) Calculated UV/Vis spectrum. B) Experimental UV/Vis spectrum (in Et_2_O) and solution of **5** (in Et_2_O). C) Frontier orbitals of compound **5**.

Compound **5** can be considered as alumaborane (diethyl ether adduct) with a naphthalene bridged Al−B σ‐bond, in which the aluminum center is four‐coordinated due to the occurrence of an additional diethyl ether donor ligand. To the best of our knowledge the reductive Al−B σ‐bond formation with aryl migration as demonstrated for **5** is the first example. As mentioned above, Al^I^ nucleophiles were employed to establish Al→B or Al−B bonds in the past, but the involvement of a transient Al^I^ species in our approach is not evident. An alternative mechanism would include the formation of an Al−B one or two electron bond prior to the elimination of the iodide. Experiments with a reduced amount of potassium graphite (1.05 eq.) to trap possible (radical) intermediates only gave mixtures of **4** and **5**. Currently the actual mechanism of this transformation remains unclear, and the elucidation proved difficult for such a type of an intramolecular process. Diethyl ether was the preferred solvent of choice. Reduction in hydrocarbons (toluene, benzene) or diisopropyl ether did not afford reasonable products. We did not find any indication for a dissociation of the coordinated diethyl ether entity in **5**, e.g. in solution, by heating (60 °C) or *in vacuo* (solid sample, 24 h). As determined by X‐ray crystallographic analysis, the bond length Al−B [2.148(2) Å] in **5** is among those of reported examples **E−G** [2.119(3)–2.156(2) Å] and shorter than the respective bond length in **H** [2.191(2) Å] (Scheme 3). The ^1^H NMR spectrum of **5** shows pseudosymmetry for the boron bound mesityl entity due to unhindered rotation around the B−C11 axis and the expected asymmetry for the aluminum bound mesityl group. A very broad signal for the ^11^B nucleus was observed at 121.3 ppm (ω_1/2_=1621 Hz), which resembles the signal observed for the boron nucleus in **H** at 109 ppm. For further insight into the nature of the Al−B σ‐bond DFT calculations were performed using (PCM: Et_2_O) *w*B97XD/6‐311++g**//*w*B97XD/6‐31g** (detailed description of computational methods and results is reported in the Supporting Information).^[14]^ The experimental bond length Al−B [2.148(2) Å] is in excellent agreement with the computed distance [2.142 Å]. The Al−B σ‐bond is found in the canonical HOMO of compound **5**, to which it contributes with a proportion of ca. 37 % (Scheme 4).^[15]^ The Wiberg index (WBI)[^15a^, ^16^] of the Al1−B1 σ‐bond (0.68) resembles the WBI indices found for the bonds Al1−C9 (0.73) and Al1−C20 (0.75). WBI values<1 for these bonds and the positive partial charge (+0.60) at the aluminum center indicate polarized bonds. For insight into the color of compound **5** experimental UV/Vis spectra in Et_2_O were recorded, which display two weak bands in the visible region at 425 nm and 520 nm (Scheme 4). TD‐DFT^[17]^ simulated UV/Vis spectra (PCM: Et_2_O *w*B97X‐D/6‐31g**) suggest one broad and weak band (oscillator strength, *f*_osc_ ≈0.01) at 466 nm (543 nm when optimization of the geometry of the excited state was performed), which we find to be in good agreement with the experimental value of 520 nm corresponding to the magenta color. The major part of the computed band is due to the formation of the first excited singlet state S1, which can be traced back to a HOMO→LUMO transition in a simplified picture (Scheme 4). While the HOMO contains contributions of the Al−B σ‐bond and the π‐orbitals of the boron bound mesityl group, the LUMO is composed of the vacant p‐orbital at boron and π*‐orbitals of the naphthalene entity. The orthogonal HOMO to LUMO alignment accounts for the low absorptivity and oscillator strength observed in compound **5**.

For insight into chemical reactivity, the Al−B σ‐bond dissociation energy Δ*E* in **5** was calculated by scanning the potential energy surface at *w*B97X‐D/6‐31g** level of theory. The calculation was complicated by the fact that the naphthalene scaffold bridges the Al−B motif, which impedes a free dissociation process. Therefore, the naphthalene entity was truncated by two phenyl entities to obtain the model compound (Mes)PhB−Al(Mes)Ph(OEt_2_), for which the dissociation at the Al−B σ‐bond was simulated (see Figure S27). Thus, the bond dissociation energy along the Al−B motif was found to be Δ*E*=382 kJ mol^−1^. The Al−B bond is stronger than the Al−C bond in AlMe_3_ with Δ*E*=264 kJ mol^−1^. In terms of the dissociation energy, the Al−B σ‐bond is comparable with the C−B σ‐bonds in BMe_3_ (Δ*E*=368 kJ mol^−1^).^[18]^ For comparison we calculated Δ*E* in the cyclopentadienyl‐boron(I)→AlI_3_ complex reported by Braunschweig,^[9]^ and obtained a lower value of Δ*E*=326 kJ mol^−1^, in accordance with the respective σ‐dative character.

**Scheme 5:**
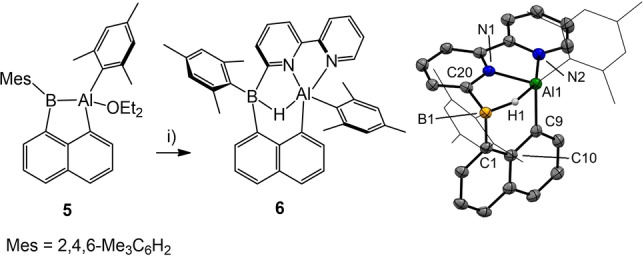
Synthesis of compound **6**. Reagents and conditions. i) 1.0 eq. 2,2′‐bipy, benzene, rt, overnight, isolated crystals. Molecular structure of compound **6** with omitted hydrogen atoms except for the bridging hydrogen atom H1. Thermal ellipsoids are presented at 50 % probability. Co‐crystallized benzene molecules are omitted. Selected bond lengths in Å and bond angles in deg. Al1−N1 1.940(3), Al1−N2 2.068(3), Al1−C9 1.985(4), C1−B1 1.625(6), C20−B1 1.626(5), Al1−H1 1.91(3), B1−H1 1.23(3), N1−Al1−N2 78.75(12), C1− B1−C20 101.0(3), C1−B1−H1 106.0(16), C20−B1−H1 102.9(2), C11−B1−H1 106.5(2).

In initial attempts to probe the chemistry of alumaborane **5** we focused on reactions with archetypal donor ligands, for which either ligand addition (to boron) or exchange of coordinated diethyl ether (at aluminum) can be expected. With this in mind, compound **5** was reacted with amines (triethyl amine, pyridine) and phosphines (trimethyl or triphenyl phosphine). ^1^H and ^11^B{^1^H} NMR monitoring indicated an initial adduct formation with **5**. However, attempts to crystallize the products only led to decomposition as assessed by NMR monitoring. Only the reaction of 2,2′‐bipyridine as a chelating donor ligand with **5** afforded crystals of compound **6** suitable for X‐ray crystallography (Scheme 5). In **6** the five‐coordinated aluminum center is chelated via two nitrogen atoms of the former 2,2′‐bipyridine entity. The formation of **6** involves the scission of the Al−B σ‐bond with concomitant insertion into the C−H aryl bond at the 3‐position of the coordinated 2,2′‐bipyridine unit. The C−H aryl bound hydrogen atom occurs as a bridging atom between aluminum and boron in the final product **6**. The reactions of **5** with donor ligands appear to be poorly predictable. Although ligand addition or exchange at boron or aluminum can be anticipated, these transformations were difficult to control at this stage and will be subject of the future investigation.

**Scheme 6:**
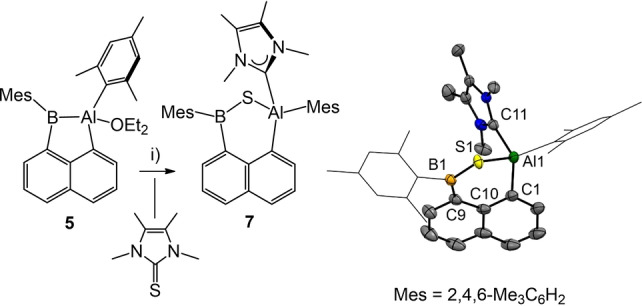
Synthesis of compound **7**. Reagents and conditions. i) 1.05 eq. S=C(NMeCMe)_2_, toluene, rt, overnight, 74 %. Molecular structure of compound **7** with omitted hydrogen atoms and thermal ellipsoids presented at 50 % probability. Selected bond lengths in Å and bond angles in deg. S1−B1 1.797(2), S1−Al1 2.2664(6), Al1−N1 1.940(3), Al1−C1 1.966(2), Al1−C(11) 2.0622(19), C9−B1 1.580(3), B1−S1−Al(1) 96.58(7), C1−Al1−C11 109.16(8), C1−Al1−S1 103.56(6), C11−Al1−S1 98.19(5), C9−B1−S1 128.83(14). Mes=2,4,6‐Me_3_C_6_H_2_.

For further investigation on the reactivity, compound **5** was reacted with substrates containing polarized multiple bonds. Thus, the reaction of **5** with tetramethyl imidazoline‐2‐thione containing a C=S bond undergoes Al−B bond scission (Scheme 6). Compound **7** was unambiguously identified by X‐ray crystallography and displays a bridging sulfur atom and an aluminum coordinated *N*‐heterocyclic carbene ligand (NHC), i.e. a tetramethyl imidazoline‐2‐ylidene entity. The reaction can be viewed as a reductive C=S bond cleavage of thione by the Al−B bond with final trapping of the scission products, i.e. sulfur and NHC. The formation of the NHC entity in **7** can be rationalized by the fact that the thione employed is a widely used precursor for the free carbene (tetramethyl imidazoline‐2‐ylidene) with strong reducing agents, e.g. potassium, in a desulfurization reaction.^[19]^ Therefore, to some extent the formation of **7** provides evidence of a strong inherent reducing potential of the Al−B bond motif. To the best of our knowledge such type of desulfurization has never been reported for the related Al−Al or B−B single bonds. The ^1^H NMR spectrum of **7** shows pseudosymmetry of the boron bound mesityl group and the NHC entity, which indicates fast rotation of these moieties around the B−C or Al−C bond axis in solution. In contrast, the aluminum bound mesityl group retains separated signals. The ^11^B{^1^H} NMR signal at 70.6 ppm (ω_1/2_=973 Hz) is in accordance with the reported boranes of type Ar_2_BSR from 70–75 ppm.^[20]^

**Scheme 7:**
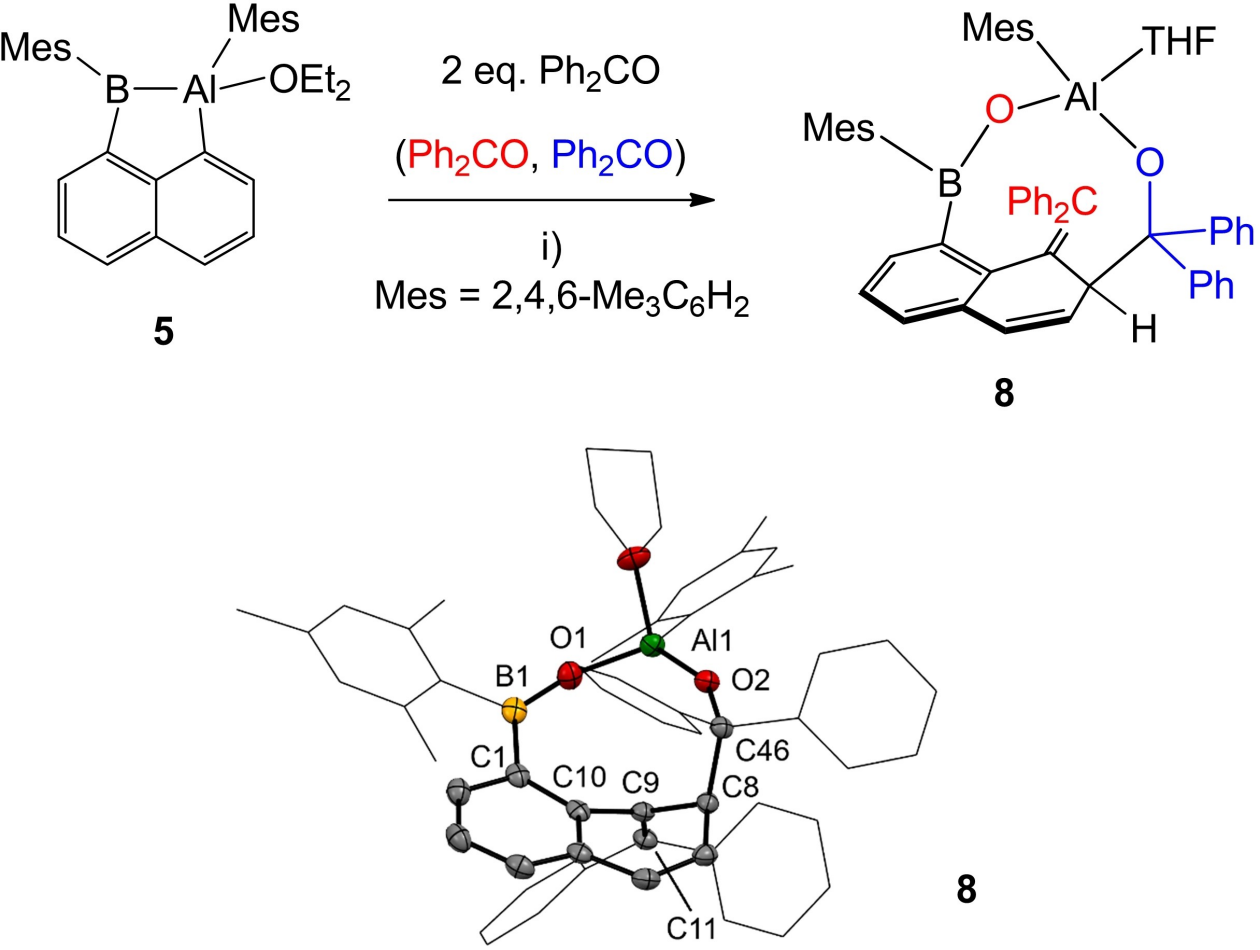
Synthesis of compound **8**. Reagents and conditions. i) 2.05 eq. benzophenone, THF, rt, overnight, 90 %. Molecular structure of compound **8** with omitted hydrogen atoms and thermal ellipsoids presented at 50 % probability. Selected bond lengths in Å and bond angles in deg. Al1−O2 1.6963(13), Al1−O1 1.7229(14), Al1−O3 1.9163(15), O1−B1 1.336(2) C1−C10 1.412(2), C1−B1 1.583(3), C8−C9 1.522(2), C9−C10 1.489(2), C9−C11 1.352(2), C8−C46 1.581(2), O2−Al1−O1 118.01(7), B1−O1−Al1 158.6(2), O1−B1−C1 122.57(16), O1−B1−C1 122.57(16), O1−B1−C24 118.41(17), C1−B1−C24 118.56(16). Mes=2,4,6‐Me_3_C_6_H_2_.

The reductive scission of C=S bonds stimulated our interest in the reactivity of other polar multiple bonds. Thus, the reaction of compound **5** with benzophenone containing a polar C=O entity was studied (Scheme 7). Reactions reproducibly necessitated 2 eq. of benzophenone and ultimately afforded compound **8**, which was identified by X‐ray crystallographic analysis. Lower stoichiometric amounts of benzophenone to elucidate the potential mechanism only afforded mixtures of **5** and **8**. In particular, we were unable to trap potential intermediates for the complex formation of **8** despite intense effort. While the aluminum coordinated THF ligand in **8** obviously results from the solvent, the formation of the final scaffold is extremely complex and can best be viewed as a stepwise separate reaction of benzophenone with the Al−B and Al−C bonds, respectively. One equivalent of benzophenone (red) may react at the Al−B motif. The formation of the Al−O−B entity in **8** can be rationalized by the strong oxophilic character of both elements and leads to a complete deoxygenation of 1 eq. of benzophenone. The formation of the exocyclic olefin fragment=CPh_2_ results from deoxygenated benzophenone. The mechanistic details of this process remain unclear at the moment due to the lack of experimentally confirmed intermediates. The second equivalent of benzophenone (blue) formally inserts into an Al−C bond with ring expansion, but complete C−O bond rupture is avoided in this case. The latter reaction step corresponds to the well‐known addition of aluminum organyls to polar C=O bonds.^[1]^ Thus, the reactivity of compound **5** toward the C=O bond in benzophenone provides a rare showcase example, in which the Al−B vs. Al−C bond can be studied in the same molecule **8**. While reductive C=O deoxy‐genation is found for the Al−B motif, addition to the C=O bond is observed for the related Al−C entity. Compound **8** was found to have limited solubility even in dichloromethane, while more polar solvents, including dimethylsulfoxide, dimethylformamide or acetonitrile, led to decomposition. The ^11^B{^1^H} NMR spectrum showed a broad singlet at 42.1 (ω_1/2_=1421 Hz), which is well comparable to the reported esters of borinic acids of type Ar_2_BOR resonating from 40–45 ppm.^[21]^

**Scheme 8:**
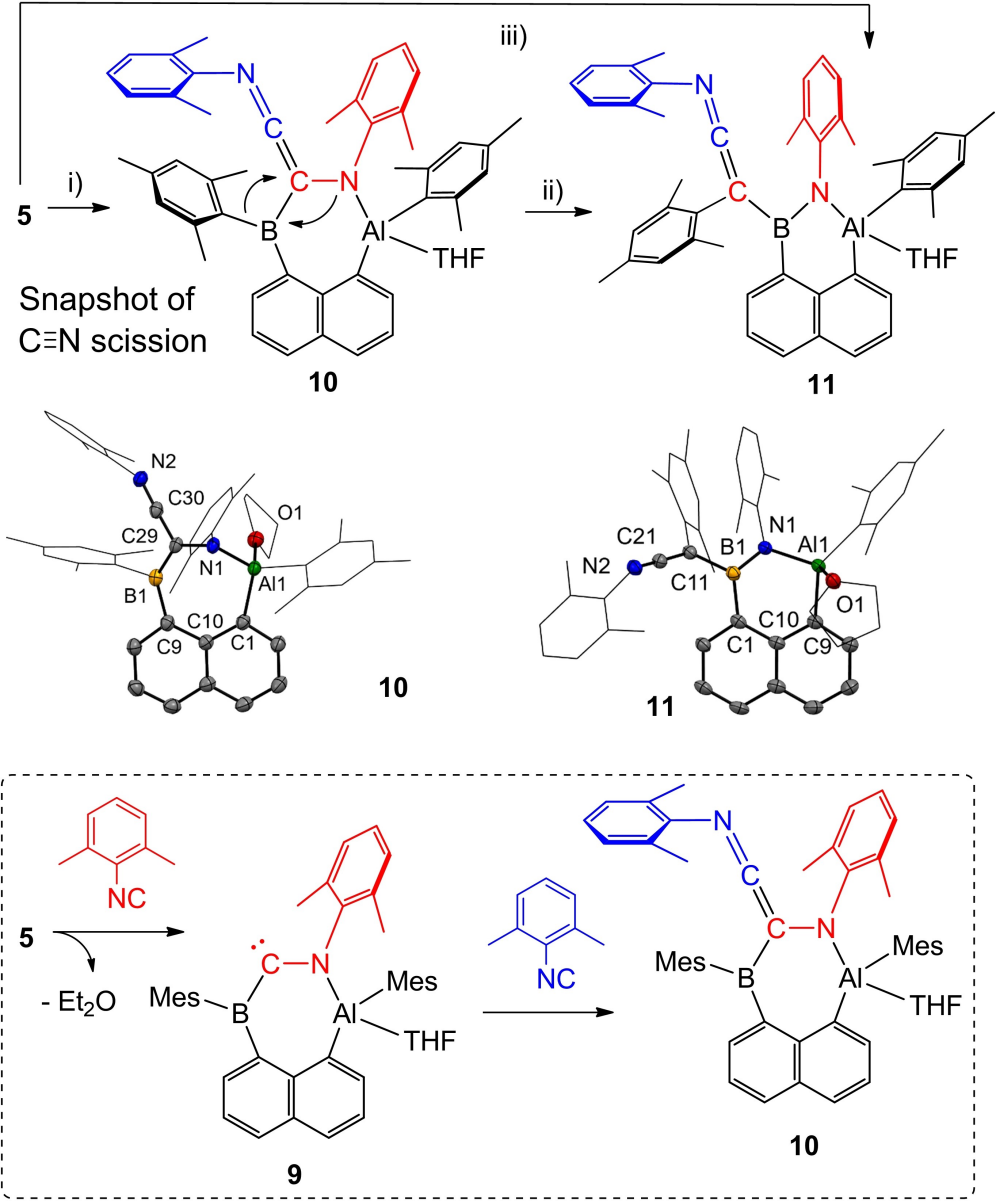
Synthesis of compound **10** and **11**. Reagents and conditions. i) 2.05 eq. 2,6‐xylyl isonitrile, THF, rt, 5 min, then crystallization, ca. 30 min, 71 %. ii) THF, rt, 24 h, 83 %. iii) 2.05 eq. 2,6‐xylyl isonitrile, THF, rt, 24 h, 83 %. THF=tetrahydrofuran. A transient intermediate **9** is proposed for the synthesis of **10**. Molecular structures of compounds **10** and **11** with omitted hydrogen atoms and thermal ellipsoids presented at 50 % probability. Selected bond lengths in Å. For **10**: Al1−N1 1.8144(15), Al1−O1 1.9229(14), Al1−C1 1.9780(18), N1−C29 1.449(2), N2−C30 1.198(2), N2−C31 1.422(2), C9−B1 1.593(3), C29−B1 1.524(3). For **11**: Al1−N1 1.8516(15), Al1−O1 1.9440(14), N1−B1 1.400(2), N2−C21 1.223(2), C11−C21 1.313(2), C11−B1 1.622(2), C1−B1 1.600(3).

In consistent extension of the reductive scission observed with C=O and C=S double bonds, we investigated the reactivity of compound **5** with 2,6‐xylyl isonitrile with the C≡N triple bond motif (Scheme 8). Again, the reaction in THF reproducibly required 2 eq. of isonitrile and afforded product **10** after 5 min and rapid crystallization. Compound **10** was obtained in the form of orange needles and unambiguously identified by X‐ray crystallography. While one of the isonitrile entities (red) underwent insertion into the Al−B bond with decrease of the bond order to a C−N single bond, the second isonitrile unit (blue) was terminally coupled with C=C double bond formation. The key motif in **10** is the seven‐membered Al,B,N‐containing heterocyclic structure, which is essentially stabilized by the disubstituted naphthalene scaffold. Compound **10** was found to be stable as a solid for at least one month, but in solution at ambient temperature (24 h) it converts into compound **11**, the identity of which was elucidated by X‐ray crystallography. The intramolecular rearrangement **10**→**11** involves ring contraction to a more stable six‐membered Al,B,N‐heterocycle and is accompanied by the formation of a thermodynamically stable B−N bond, as well as by a (formal) 1,2‐migration process of the boron bound mesityl entity to the adjacent carbon atom. In the event, the C−N single bond in **10** (red) is ultimately cleaved. Therefore, compound **11** can be viewed as the final product of a complete C≡N triple bond scission in the isonitrile by alumaborane **5**, and compound **10** represents an intermediate (snapshot) on the pathway toward the final compound **11**. The NMR spectroscopic characterization of **10** was performed at −60 °C in order to freeze the intramolecular formation of **11**. Although no diagnostic ^11^B{^1^H} signal was detected for **10** (probably due to the asymmetric environment at boron), a broad signal at 45.0 ppm (ω_1/2_=1200 Hz) was observed for **11**, which compares well with aminoboranes of type Ar_2_BNR_2_ with resonances from 40–45 ppm.^[22]^

The observed terminal coupling of the isonitrile entities in combination with C≡N triple bond scission by the Al−B single bond is unique. For related Al−Al single bonds reactions with isocyanides did not show any C≡N triple bond scission in past and formed products of different nature than reported herein.^[23]^ Only one example of a C≡N triple bond scission by a B−B single bond motif is well documented by Yamashita.^[24]^ We then investigated the conversion of **5** into **11** via the isolated intermediate **10** by means of DFT calculations (PCM: THF *w*B97X‐D/6‐311++g**//*w*B97X‐D/6‐31g**, see Supporting Information and Figure S28). As expected for the formation of **11** from **5** the overall reaction was found to be highly exergonic as indicated by the Gibbs free energy change of Δ*G*°=−405 kJ mol^−1^. Intermediate **10** is formed from **5** in a strong downhill process (Δ*G*°=−256 kJ mol^−1^), a thermodynamic stabilization that accounts for the slow decomposition of this species. For the formation of **10** a transient (amino boryl) carbene species **9** is supposed, which is supported by DFT calculations (structure IV in Figure S28). Compound **9** is reasonably formed from **5** with scission of the Al−B bond and ring expansion from a five‐ to a seven‐membered cycle. The addition of a second equivalent of isonitrile to the distinct carbon atom in **9** affords the observed intermediate **10**. The low activation barrier of 52 kJ mol^−1^ to form **10** agrees well with the rapid formation (5 min) and fast crystallization observed under laboratory conditions. The conversion of **10** into the final product **11** is mainly characterized by a mesityl 1,2‐migration from boron to the C=C bond (Scheme 8, and Figure S28). Our calculations strongly support a reaction mechanism, in which the mesityl entity is first transferred to the central carbon atom (C30) of the ethenimine fragment. The calculated energy barrier related to this indirect mesityl migration is 100.4 kJ mol^−1^. Assuming a DFT error of ±8 kJ mol^−1^, this energy barrier can be overcome at ambient temperature, which explains the spontaneous conversion of **10** into **11** after 24 h in solution. A lower‐energy (<37 kJ mol^−1^) subsequent mesityl migration toward the neighboring C atom moiety occurs simultaneously with the formation of the B−N bond to yield **11** in an energy‐downhill process. It is worth mentioning that the B−N bond in **11** is characterized by π‐bonding orbitals. In fact, we calculated a WBI value of 1.50 and the N1−B1 bond length of 1.400(2) Å (DFT 1.404 Å) obtained from the crystal structure suggest the double‐bond character of the B−N fragment.^[25]^ The formation of the B−N bond with significant π‐contribution accounts for the thermodynamically favored conversion of **10** into **11**. Remarkably the direct 1,2‐migration of the mesityl entity to C29 in **10** could not be calculated on a reasonable basis. A single‐step mesityl transfer would involve C−N bond cleavage, heterocycle contraction, and B=N bond formation. On the basis of Hammond's postulate,^[26]^ the direct formation of **11** through single‐step mesityl 1,2‐migration is less plausible due to the high exergonicity of the reaction of 405.0 kJ mol^−1^ (see Supporting Information).

## Conclusion

In conclusion, we have elaborated a reliable synthesis of alumaborane **5** in the form of its diethyl ether adduct, which contains a rare example of an electron precise Al−B σ‐bond. The *peri*‐disubstituted naphthalene scaffold was found to be essential due to the pronounced preorientation of the aluminyl and boryl entities in precursors **3** and **4**, which show remarkable contact interactions. Access to **5** is facilitated by an unprecedented intramolecular reductive rearrangement in **4**, which involves migration of a mesityl entity from boron to aluminum and Al−B σ‐bond formation. While adduct formation at the Lewis acidic boron or aluminum centers proved to be difficult, compound **5** readily reacted with thermodynamically stable polarized multiple bonds, i.e. C=S, C=O, and C≡N. The respective final products **7**, **8**, and **11** showed complete rupture of these bonds with neat insertion of the heteroatom into the Al−B bond and incorporation of the de‐elemented fragment into the resulting product. This reactivity of the Al−B bond is in contrast to Al−C bonds in organometallic reagents, for which commonly addition reactions to these motifs are observed. The reactivity of **5** towards unpolarized multiple bonds, i.e. C=C and C≡C in (internal and terminal) alkenes and alkynes, was also investigated. No reaction was observed for all cases and the starting materials were recovered.

## Experimental Section

Synthesis details, NMR spectra, crystallographic data, and detailed computational investigations and optimized Cartesian coordinates (PDF) are provided in the section Supporting Information, which is available free of charge. For CCDC depository numbers see ref. [27].

## Conflict of interest

The authors declare no conflict of interest.

1

## Data Availability

The data that support the findings of this study are available from the corresponding author upon reasonable request.
